# 4-Nitro­phenyl 4-bromo­benzene­sulfonate

**DOI:** 10.1107/S1600536809040033

**Published:** 2009-10-10

**Authors:** Nagarajan Vembu, Frank R. Fronczek

**Affiliations:** a Department of Chemistry, Urumu Dhanalakshmi College, Tiruchirappalli 620 019, India; b Department of Chemistry, Louisiana State University, Baton Rouge, LA 70803-1804, USA

## Abstract

In the title mol­ecule, C_12_H_8_BrNO_5_S, the dihedral angle between the two benzene rings is 30.02 (7)°. The crystal structure is stabilized by weak C—H⋯O inter­actions.

## Related literature

For a detailed account of the mol­ecular and supra­molecular architectures of aromatic sulfonates, see: Vembu *et al.* (2007 ▶) and references cited therein. For the uses of aromatic sulfonates, see: Alford *et al.* (1991 ▶); Jiang *et al.* (1990 ▶); Narayanan & Krakow (1983 ▶); Spungin *et al.* (1992 ▶); Tharakan *et al.* (1992 ▶); Yachi *et al.* (1989 ▶). For C—H⋯O inter­actions, see: Desiraju & Steiner (1999 ▶). For hydrogen-bond motifs, see: Bernstein *et al.* (1995 ▶).
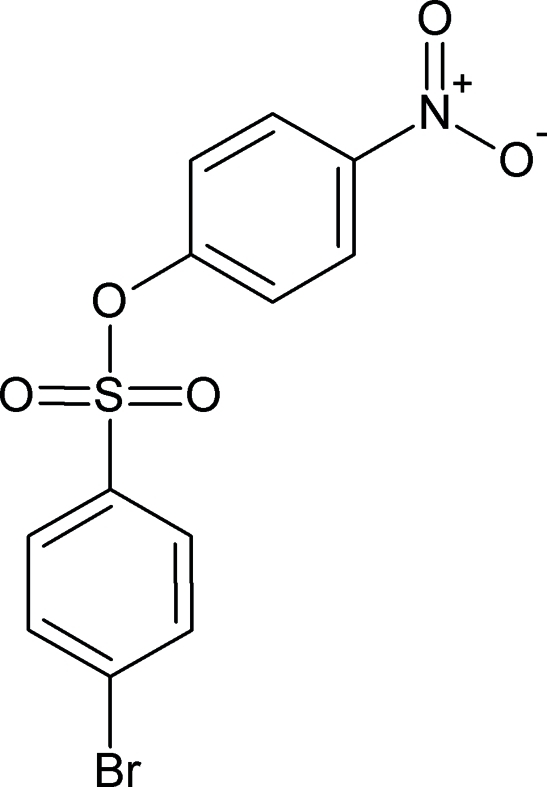


## Experimental

### 

#### Crystal data

C_12_H_8_BrNO_5_S
*M* *_r_* = 358.16
Monoclinic, 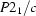

*a* = 13.150 (2) Å
*b* = 8.3387 (10) Å
*c* = 12.292 (2) Å
β = 105.932 (7)°
*V* = 1296.1 (3) Å^3^
*Z* = 4
Mo *K*α radiation
μ = 3.35 mm^−1^
*T* = 90 K
0.20 × 0.15 × 0.07 mm

#### Data collection

Nonius KappaCCD diffractometer with an Oxford Cryosystems Cryostream cooler
Absorption correction: multi-scan (*SCALEPACK*; Otwinowski & Minor, 1997 ▶) *T*_min_ = 0.554, *T*_max_ = 0.799
35540 measured reflections
4458 independent reflections
3518 reflections with *I* > 2σ(*I*)
*R*_int_ = 0.024

#### Refinement

*R*[*F* ^2^ > 2σ(*F* ^2^)] = 0.036
*wR*(*F* ^2^) = 0.085
*S* = 1.04
4458 reflections
213 parameters
All H-atom parameters refined
Δρ_max_ = 0.57 e Å^−3^
Δρ_min_ = −0.81 e Å^−3^

### 

Data collection: *COLLECT* (Nonius, 2000 ▶); cell refinement: *DENZO* and *SCALEPACK* (Otwinowski & Minor, 1997 ▶); data reduction: *DENZO* and *SCALEPACK*; program(s) used to solve structure: *SHELXS97* (Sheldrick, 2008 ▶); program(s) used to refine structure: *SHELXL97* (Sheldrick, 2008 ▶); molecular graphics: *PLATON* (Spek, 2009 ▶); software used to prepare material for publication: *SHELXL97*.

## Supplementary Material

Crystal structure: contains datablocks I, global. DOI: 10.1107/S1600536809040033/lh2921sup1.cif

Structure factors: contains datablocks I. DOI: 10.1107/S1600536809040033/lh2921Isup2.hkl

Additional supplementary materials:  crystallographic information; 3D view; checkCIF report

## Figures and Tables

**Table 1:** Hydrogen-bond geometry (Å, °)

*D*—H⋯*A*	*D*—H	H⋯*A*	*D*⋯*A*	*D*—H⋯*A*
C3—H3⋯O8	0.92 (3)	2.55 (3)	2.930 (3)	105.0 (19)
C11—H11⋯O8^i^	0.96 (3)	2.42 (3)	3.288 (3)	150 (2)
C15—H15⋯O7^ii^	0.98 (3)	2.42 (3)	3.282 (3)	146 (2)

Symmetry codes: (i) 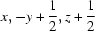; (ii) 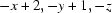.

**Fig. 1.**
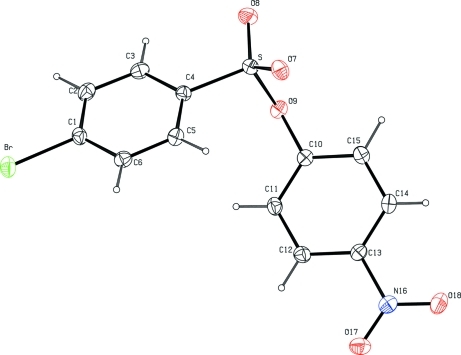
The asymmetric unit of (I) with the atoms labelled and displacement ellipsoids depicted at the 50% probability level for all non-H atoms. H-atoms are drawn as spheres of arbitrary radius

**Fig. 2.**
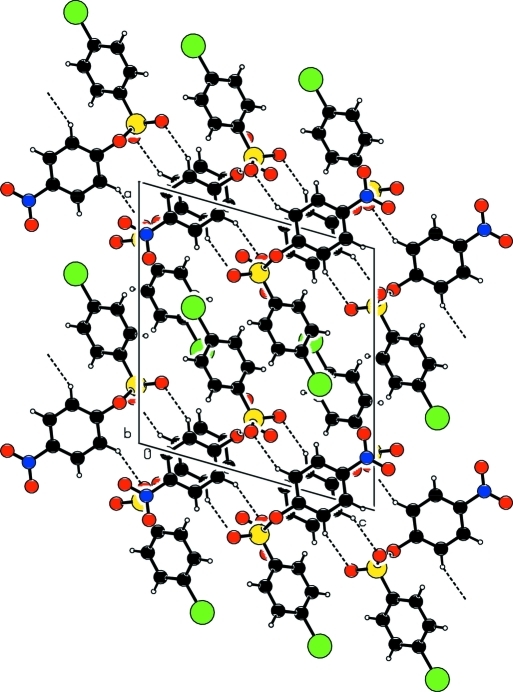
The molecular packing viewed down the b-axis. Dashed lines represent the weak C—H···O interactions within the lattice.

## supplementary crystallographic information

### Comment

Aromatic sulfonates are used in monitoring the merging of lipids (Yachi *et al.*, 1989) and in many other fields (Spungin *et al.*, 1992; Tharakan *et al.*,1992; Alford *et al.*, 1991; Jiang *et al.*, 1990; Narayanan & Krakow, 1983). An X-ray study of the title compound was undertaken in order to determine its crystal and molecular structure owing to the biological importance of its analogues. The molecular structure of (I) is shown in Fig. 1. The S—C, S—O and S=O bond lengths are comparable with those found in related structures which have been previously reported by us (Vembu *et al.* 2007 and references cited therein).

The C4–S–O9–C10 torsion angle of -86.5 (2)° corresponds to -synclinal conformation; as expected the dihedral angle between the mean planes of the nitrophenyl and bromobenzene rings of 30.02 (7)° shows that the two rings are not coplanar. This is similar to the situation reported by us for other aromatic sulfonates (Vembu *et al.* 2007 and references cited therein)

The crystal structure of (I) is stabilized by weak intermolecular C—H···O interactions (Desiraju *et al.*, 1999) (Table 1, Fig. 2). Two symmetry related C15–H15···O7^ii^ interactions generate a binary motif of graph set, *R*^2^_2_(12) (Bernstein *et al.*,  1995).

### Experimental

4-Bromobenzenesulfonyl chloride (10 mmol), dissolved in acetone (10 ml), was added dropwise to 4-Nitrophenol (10 mmol) in aqueous NaOH (8 ml, 5%) with constant stirring. The precipitate (6.5 mmol, yield 65%) was filtered and recrystallized from aqueous ethanol.

### Refinement

All H-atoms were located in difference maps and their positions and isotropic displacement parameters freely refined.

### Crystal data

**Table:**

C_12_H_8_BrNO_5_S	*F*(000) = 712
*M**_r_* = 358.16	*D*_x_ = 1.836 Mg m^−^^3^
Monoclinic, *P*2_1_/*c*	Melting point: 376 K
Hall symbol: -P 2ybc	Mo *K*α radiation, λ = 0.71073 Å
*a* = 13.150 (2) Å	Cell parameters from 4455 reflections
*b* = 8.3387 (10) Å	θ = 2.5–32.6°
*c* = 12.292 (2) Å	µ = 3.35 mm^−^^1^
β = 105.932 (7)°	*T* = 90 K
*V* = 1296.1 (3) Å^3^	Plate, colorless
*Z* = 4	0.20 × 0.15 × 0.07 mm

### Data collection

**Table:**

Nonius KappaCCD diffractometer with an Oxford Cryosystems Cryostream cooler	4458 independent reflections
Radiation source: fine-focus sealed tube	3518 reflections with *I* > 2σ(*I*)
graphite	*R*_int_ = 0.024
ω scans with κ offsets	θ_max_ = 32.6°, θ_min_ = 2.9°
Absorption correction: multi-scan (*SCALEPACK*; Otwinowski & Minor, 1997)	*h* = −19→19
*T*_min_ = 0.554, *T*_max_ = 0.799	*k* = −12→12
35540 measured reflections	*l* = −17→18

### Refinement

**Table:**

Refinement on *F*^2^	Primary atom site location: structure-invariant direct methods
Least-squares matrix: full	Secondary atom site location: difference Fourier map
*R*[*F*^2^ > 2σ(*F*^2^)] = 0.036	Hydrogen site location: inferred from neighbouring sites
*wR*(*F*^2^) = 0.085	All H-atom parameters refined
*S* = 1.03	*w* = 1/[σ^2^(*F*_o_^2^) + (0.0315*P*)^2^ + 2.0753*P*] where *P* = (*F*_o_^2^ + 2*F*_c_^2^)/3
4458 reflections	(Δ/σ)_max_ = 0.001
213 parameters	Δρ_max_ = 0.57 e Å^−^^3^
0 restraints	Δρ_min_ = −0.81 e Å^−^^3^

### Special details

**Table:**

Geometry. All e.s.d.'s (except the e.s.d. in the dihedral angle between two l.s. planes) are estimated using the full covariance matrix. The cell e.s.d.'s are taken into account individually in the estimation of e.s.d.'s in distances, angles and torsion angles; correlations between e.s.d.'s in cell parameters are only used when they are defined by crystal symmetry. An approximate (isotropic) treatment of cell e.s.d.'s is used for estimating e.s.d.'s involving l.s. planes.
Refinement. Refinement of *F*^2^ against ALL reflections. The weighted *R*-factor *wR* and goodness of fit *S* are based on *F*^2^, conventional *R*-factors *R* are based on *F*, with *F* set to zero for negative *F*^2^. The threshold expression of *F*^2^ > σ(*F*^2^) is used only for calculating *R*-factors(gt) *etc*. and is not relevant to the choice of reflections for refinement. *R*-factors based on *F*^2^ are statistically about twice as large as those based on *F*, and *R*- factors based on ALL data will be even larger.

### Fractional atomic coordinates and isotropic or equivalent isotropic displacement parameters (Å^2^)

**Table:**

	*x*	*y*	*z*	*U*_iso_*/*U*_eq_	
Br	0.421887 (17)	0.40490 (3)	0.26901 (2)	0.02131 (7)	
S	0.78218 (4)	0.39271 (6)	0.01144 (4)	0.01524 (10)	
C1	0.52731 (16)	0.3985 (3)	0.19030 (18)	0.0161 (4)	
C2	0.51138 (17)	0.3089 (3)	0.09190 (19)	0.0190 (4)	
C3	0.59099 (17)	0.3057 (3)	0.03702 (18)	0.0182 (4)	
C4	0.68192 (16)	0.3964 (3)	0.08048 (17)	0.0150 (4)	
C5	0.69638 (16)	0.4885 (3)	0.17766 (18)	0.0162 (4)	
C6	0.61882 (17)	0.4870 (3)	0.23466 (18)	0.0172 (4)	
O7	0.83334 (13)	0.5453 (2)	0.02325 (14)	0.0205 (3)	
O8	0.74442 (13)	0.3181 (2)	−0.09633 (13)	0.0219 (3)	
O9	0.86612 (12)	0.26386 (18)	0.08300 (13)	0.0166 (3)	
C10	0.94553 (16)	0.3146 (2)	0.17970 (17)	0.0150 (4)	
C11	0.92572 (16)	0.3055 (3)	0.28444 (18)	0.0169 (4)	
C12	1.00721 (17)	0.3432 (3)	0.37986 (18)	0.0170 (4)	
C13	1.10505 (16)	0.3843 (2)	0.36629 (18)	0.0156 (4)	
C14	1.12437 (16)	0.3955 (3)	0.26150 (19)	0.0180 (4)	
C15	1.04210 (17)	0.3612 (3)	0.16568 (19)	0.0178 (4)	
N16	1.19300 (15)	0.4124 (2)	0.46863 (16)	0.0184 (3)	
O17	1.17261 (14)	0.4151 (2)	0.56006 (13)	0.0234 (3)	
O18	1.28217 (13)	0.4302 (2)	0.45684 (15)	0.0256 (4)	
H2	0.449 (2)	0.245 (4)	0.062 (2)	0.024 (7)*	
H3	0.582 (2)	0.249 (3)	−0.029 (2)	0.020 (7)*	
H5	0.758 (2)	0.552 (3)	0.205 (2)	0.019 (7)*	
H6	0.627 (2)	0.542 (3)	0.302 (2)	0.018 (7)*	
H11	0.858 (2)	0.274 (4)	0.291 (2)	0.028 (8)*	
H12	0.994 (2)	0.340 (3)	0.452 (2)	0.021 (7)*	
H14	1.188 (2)	0.425 (3)	0.254 (2)	0.022 (7)*	
H15	1.053 (2)	0.367 (4)	0.090 (2)	0.025 (7)*	

### Atomic displacement parameters (Å^2^)

**Table:**

	*U*^11^	*U*^22^	*U*^33^	*U*^12^	*U*^13^	*U*^23^
Br	0.01653 (10)	0.02210 (11)	0.02718 (12)	0.00233 (8)	0.00914 (8)	0.00332 (9)
S	0.0167 (2)	0.0152 (2)	0.0142 (2)	0.00039 (18)	0.00489 (17)	0.00036 (18)
C1	0.0145 (8)	0.0160 (9)	0.0190 (9)	0.0025 (7)	0.0067 (7)	0.0034 (8)
C2	0.0158 (9)	0.0182 (10)	0.0211 (10)	−0.0028 (8)	0.0017 (8)	0.0007 (8)
C3	0.0194 (10)	0.0171 (10)	0.0162 (9)	−0.0019 (8)	0.0019 (7)	−0.0031 (8)
C4	0.0148 (8)	0.0161 (9)	0.0145 (9)	0.0012 (7)	0.0045 (7)	0.0014 (7)
C5	0.0154 (9)	0.0139 (9)	0.0190 (10)	−0.0005 (7)	0.0042 (7)	−0.0018 (7)
C6	0.0180 (9)	0.0164 (10)	0.0163 (9)	0.0005 (8)	0.0034 (7)	−0.0015 (8)
O7	0.0228 (8)	0.0173 (7)	0.0239 (8)	−0.0018 (6)	0.0106 (6)	0.0024 (6)
O8	0.0229 (8)	0.0266 (9)	0.0164 (7)	0.0016 (6)	0.0056 (6)	−0.0027 (6)
O9	0.0157 (7)	0.0138 (7)	0.0191 (7)	−0.0006 (5)	0.0029 (5)	−0.0023 (5)
C10	0.0156 (9)	0.0127 (9)	0.0164 (9)	−0.0004 (7)	0.0037 (7)	−0.0001 (7)
C11	0.0150 (9)	0.0167 (9)	0.0204 (10)	0.0005 (8)	0.0070 (8)	0.0037 (8)
C12	0.0183 (9)	0.0163 (9)	0.0169 (9)	0.0016 (8)	0.0055 (8)	0.0035 (8)
C13	0.0150 (9)	0.0137 (9)	0.0172 (9)	0.0014 (7)	0.0028 (7)	−0.0005 (7)
C14	0.0142 (9)	0.0175 (10)	0.0231 (10)	−0.0008 (8)	0.0065 (8)	−0.0008 (8)
C15	0.0183 (9)	0.0180 (10)	0.0187 (10)	−0.0013 (8)	0.0076 (8)	−0.0009 (8)
N16	0.0188 (8)	0.0149 (8)	0.0198 (8)	0.0007 (7)	0.0026 (7)	0.0008 (7)
O17	0.0268 (8)	0.0254 (9)	0.0168 (7)	−0.0015 (7)	0.0039 (6)	−0.0015 (6)
O18	0.0157 (7)	0.0318 (10)	0.0279 (9)	−0.0007 (7)	0.0034 (6)	−0.0010 (7)

### Geometric parameters (Å, °)

**Table:**

Br—C1	1.897 (2)	O9—C10	1.414 (2)
S—O8	1.4239 (16)	C10—C15	1.383 (3)
S—O7	1.4277 (17)	C10—C11	1.384 (3)
S—O9	1.6167 (16)	C11—C12	1.390 (3)
S—C4	1.753 (2)	C11—H11	0.96 (3)
C1—C2	1.388 (3)	C12—C13	1.385 (3)
C1—C6	1.389 (3)	C12—H12	0.95 (3)
C2—C3	1.393 (3)	C13—C14	1.383 (3)
C2—H2	0.96 (3)	C13—N16	1.475 (3)
C3—C4	1.392 (3)	C14—C15	1.393 (3)
C3—H3	0.92 (3)	C14—H14	0.91 (3)
C4—C5	1.389 (3)	C15—H15	0.98 (3)
C5—C6	1.387 (3)	N16—O17	1.225 (2)
C5—H5	0.95 (3)	N16—O18	1.229 (2)
C6—H6	0.93 (3)		
			
O8—S—O7	121.22 (10)	C1—C6—H6	119.2 (17)
O8—S—O9	103.14 (9)	C10—O9—S	119.66 (13)
O7—S—O9	107.73 (9)	C15—C10—C11	123.02 (19)
O8—S—C4	109.96 (10)	C15—C10—O9	118.07 (18)
O7—S—C4	109.35 (10)	C11—C10—O9	118.77 (18)
O9—S—C4	103.88 (9)	C10—C11—C12	118.31 (19)
C2—C1—C6	122.4 (2)	C10—C11—H11	120.7 (18)
C2—C1—Br	120.21 (16)	C12—C11—H11	121.0 (18)
C6—C1—Br	117.42 (16)	C13—C12—C11	118.7 (2)
C1—C2—C3	118.6 (2)	C13—C12—H12	122.0 (17)
C1—C2—H2	122.1 (17)	C11—C12—H12	119.2 (17)
C3—C2—H2	119.2 (17)	C14—C13—C12	122.90 (19)
C4—C3—C2	119.0 (2)	C14—C13—N16	118.82 (18)
C4—C3—H3	120.2 (16)	C12—C13—N16	118.25 (19)
C2—C3—H3	120.8 (16)	C13—C14—C15	118.37 (19)
C5—C4—C3	122.1 (2)	C13—C14—H14	121.6 (18)
C5—C4—S	118.84 (16)	C15—C14—H14	120.1 (18)
C3—C4—S	119.07 (16)	C10—C15—C14	118.6 (2)
C6—C5—C4	118.91 (19)	C10—C15—H15	120.4 (17)
C6—C5—H5	120.2 (17)	C14—C15—H15	120.9 (17)
C4—C5—H5	120.9 (17)	O17—N16—O18	124.18 (19)
C5—C6—C1	119.0 (2)	O17—N16—C13	117.87 (18)
C5—C6—H6	121.8 (17)	O18—N16—C13	117.95 (18)
			
C6—C1—C2—C3	1.2 (3)	C4—S—O9—C10	−86.54 (16)
Br—C1—C2—C3	−179.34 (16)	S—O9—C10—C15	−91.9 (2)
C1—C2—C3—C4	−2.1 (3)	S—O9—C10—C11	92.2 (2)
C2—C3—C4—C5	0.8 (3)	C15—C10—C11—C12	−0.8 (3)
C2—C3—C4—S	−179.91 (17)	O9—C10—C11—C12	174.83 (19)
O8—S—C4—C5	−168.82 (17)	C10—C11—C12—C13	−1.5 (3)
O7—S—C4—C5	−33.4 (2)	C11—C12—C13—C14	2.5 (3)
O9—S—C4—C5	81.36 (18)	C11—C12—C13—N16	−175.55 (19)
O8—S—C4—C3	11.9 (2)	C12—C13—C14—C15	−1.1 (3)
O7—S—C4—C3	147.24 (17)	N16—C13—C14—C15	176.93 (19)
O9—S—C4—C3	−97.96 (18)	C11—C10—C15—C14	2.2 (3)
C3—C4—C5—C6	1.6 (3)	O9—C10—C15—C14	−173.47 (19)
S—C4—C5—C6	−177.71 (16)	C13—C14—C15—C10	−1.2 (3)
C4—C5—C6—C1	−2.5 (3)	C14—C13—N16—O17	174.0 (2)
C2—C1—C6—C5	1.2 (3)	C12—C13—N16—O17	−7.8 (3)
Br—C1—C6—C5	−178.29 (16)	C14—C13—N16—O18	−6.5 (3)
O8—S—O9—C10	158.70 (15)	C12—C13—N16—O18	171.6 (2)
O7—S—O9—C10	29.39 (17)		

### Hydrogen-bond geometry (Å, °)

**Table:**

*D*—H···*A*	*D*—H	H···*A*	*D*···*A*	*D*—H···*A*
C3—H3···O8	0.92 (3)	2.55 (3)	2.930 (3)	105.0 (19)
C11—H11···O8^i^	0.96 (3)	2.42 (3)	3.288 (3)	150 (2)
C15—H15···O7^ii^	0.98 (3)	2.42 (3)	3.282 (3)	146 (2)

Symmetry codes: (i) *x*, −*y*+1/2, *z*+1/2; (ii) −*x*+2, −*y*+1, −*z*.
